# Evolution of relict floodplain forest in river stretches of Western and Central Europe as affected by river infrastructure networks

**DOI:** 10.1371/journal.pone.0257593

**Published:** 2021-09-29

**Authors:** Jean-Nicolas Beisel, Cybill Staentzel, Grzegorz Skupinski, Anaïs Walch, Manon Pons, Sebastian Weber, Carine Granier, Andreas Huber

**Affiliations:** 1 UMR 7362 CNRS LIVE, Université de Strasbourg, Strasbourg, France; 2 Ecole Nationale du Génie de l’Eau et de l’Environnement (ENGEES), Strasbourg, France; 3 CNRS, Laboratoire Image Ville Environnement UMR 7362, Strasbourg, France; 4 European Institute for Energy Research, Karlsruhe, Germany; 5 Electricité de France (EDF)—Centre for Hydro Engineering, Paris, France; University of Bucharest, ROMANIA

## Abstract

We studied the impact of infrastructure networks on relict floodplain forest along three stretches of the Upper Rhine (Kembs-Efringen-Kirchen, Strasbourg-Kehl and Beinheim-Iffezheim) and the Inn-Danube (Mulheim-Obernberg, Passau-Ingling and Engelhartszell-Jochenstein), each on the border between two countries. We analysed land use patterns within a 500 m wide buffer area along the main channel using photo-interpretation and compared the situations between the 1950s, 1980’s and 2010’s. Temporal changes were assessed with transition matrices and selected spatial metrics, including fragmentation indices. Over this period, forest area remained similar at three sites, increased slightly at two sites and decreased at one site. However, on average, 12.5% of floodplain forest had changed location (range: 7.3% (Engelhartszell-Jochenstein)– 26.5% (Kembs-Efringen-Kirchen)). The natural development of unmanaged areas and agricultural abandonment after World War II has led to the emergence of young riparian forests along rivers. In the Upper Rhine region, the results showed asymmetry in these two factors, with unmanaged natural areas most important on the French side and agricultural abandonment on the German side. Along the Inn-Danube, agricultural abandonment has led to an increase or stagnation of floodplain forest areas. In most cases, development of transport infrastructure between the 1950s and 2010s has caused fragmentation of the forest area, reducing the relict forest to a patchy green corridor with reduced functionality and interfacing. To go further and improve the management of these relict forests, we have to investigate the interdependency between practices related to infrastructure operation and the role that biodiversity plays for stakeholders.

## Introduction

The alluvial plains of large European rivers have been heavily modified throughout the last two centuries to meet different human uses, including transport, hydropower generation, industrial production and housing. River channelization applied in the mid- and late-nineteenth century has resulted in profound landscape transformation, with multi-channel river ecosystems reduced to single-thread channel configurations. This major change has led to modifications in land use at the expense of the riparian forest that naturally surrounded large rivers and provided a smooth transition between the aquatic environment and adjacent terrestrial ecosystems [1–4]. In many cases, the natural floodplain forest (also called alluvial forest or riparian forest) has been reduced to a thin buffer of vegetation extending laterally from the residual active channel to the uplands [5]. This relict floodplain forest constitutes an essential element of the green corridor running along watercourses, and a key element of the landscape matrix. In the absence of overflow from the river due to channelization, floodplain forest sustainability is closely linked to the land use and development on the alluvial floodplain [6].

From the end of the Second World War (1945), regional development in floodplains encouraged the movement of people and goods within economically dynamic areas along large rivers such as the Rhine. While developments during this period are important for explaining the current situation, few studies have evaluated recent changes in large river floodplain forests, particularly in the face of developing infrastructure networks. A singular socio-ecosystem case relates to river stretches that constitute the border between two countries, where management and development may differ on each side of the river. Nowadays, the interdependency between infrastructures operation and usage and the relict alluvial forests need to be investigated in order to improve the conservation of these patrimonial areas of high biodiversity.

Here, we examine temporal changes in relict floodplain forests along the Upper Rhine River and the Danube, focussing on two reaches representing the borders between (1) France and Germany (Rhine) and (2) Austria and Germany (Danube and its tributary, the Inn). The Rhine and Danube are the largest rivers in Western and Central Europe, and both are characterized by warm-season high waters that create favourable conditions for vegetation and important seasonal variations in discharge that result in bed instability [7]. These two fluvial stretches have been strongly shaped by infrastructures such as channelization, hydropower plants, locks, railway crossings and cycling paths [Rhine: 8, 9; Danube: 10, 11]. The Upper Rhine and the Danube are the oldest and most important trade routes in Europe. While such infrastructure developments have boosted regional economic development over the last two centuries, they also have the potential for negative impacts on aquatic and terrestrial biodiversity, especially by interrupting ecological continuity and reducing floodplain forest and aquatic wetlands. Our aims in this study were to (i) analyse land use changes along the Upper Rhine and Inn-Danube rivers since the 1950s using both global analyses and separate analyses for each side of the river, (ii) quantify the level to which densification of linear transport and energy infrastructure networks has co-occurred with changes in land use, and (iii) evaluate the level of relict floodplain forest fragmentation resulting from past land use planning and infrastructure network development.

## Material and methods

### Study sites

**Fig 1:**
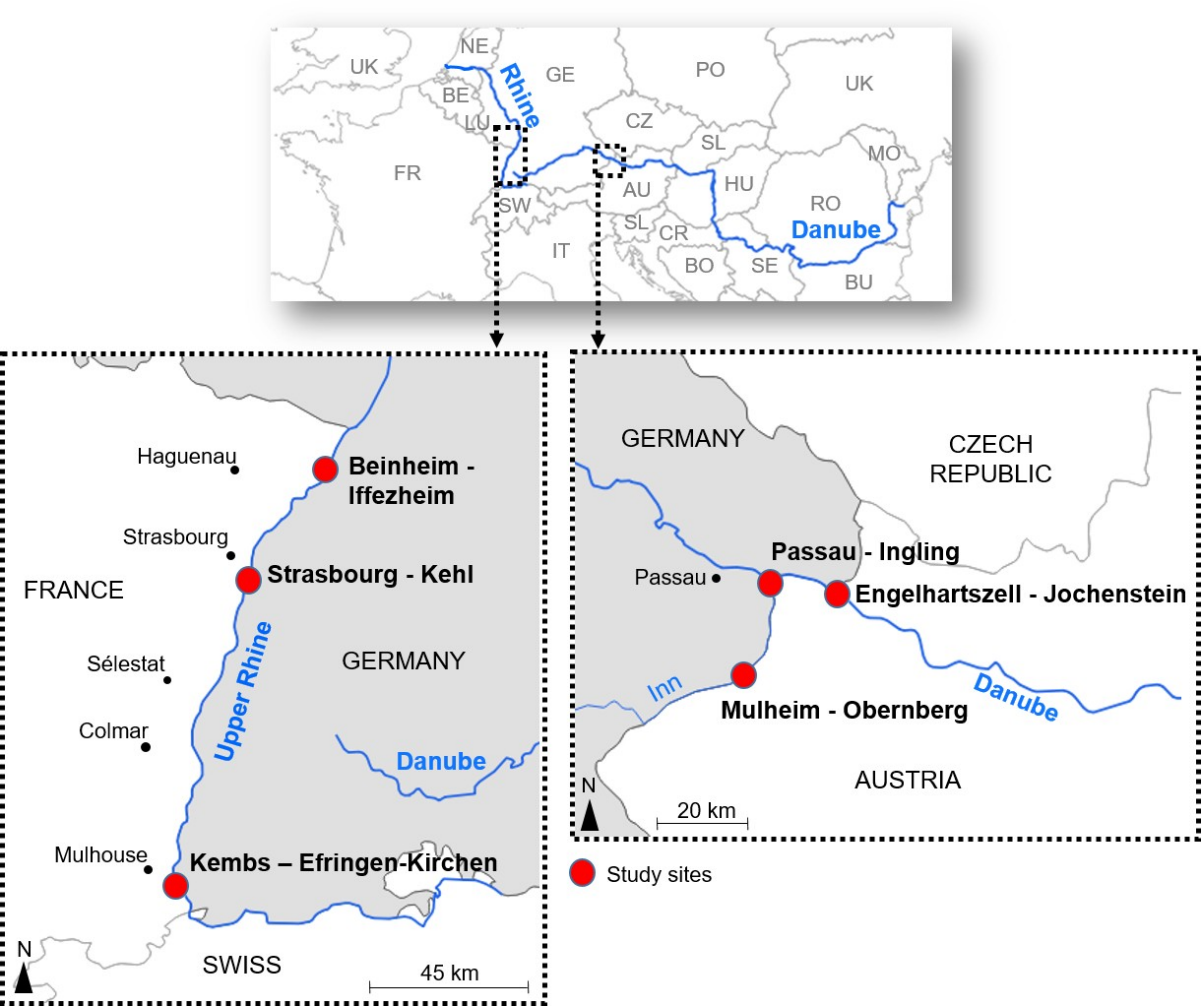
Location of study sites along the Upper Rhine (Kembs-Efringen-Kirchen, Strasbourg-Kehl and Beinheim-Iffezheim) and at the confluence of the Inn (Mulheim-Obernberg and Passau-Ingling) and Danube (Engelhartszell-Jochenstein).

Our study was based on three sites along the Upper Rhine and three sites along the Inn-Danube rivers (Fig 1) using data covering the 1950s, 1980s and 2010s. For each river, we retained one site close to an urban area (Strasbourg-Kehl, Passau-Ingling), one in a protected environment within a Natura 2000 area (Kembs-Efringen-Kirchen, Mulheim-Obernberg) and one bordered by a rural area (Beinheim-Iffezheim, Engelhartszell-Jochenstein). Each study area was defined according to the position of a hydroelectric dam, framed with a minimum length of 1 km upstream and downstream to take account of road or pedestrian bridges in relationship with the infrastructure.

The sites on the Upper Rhine were situated around Kembs (kilometric point 174, kp hereafter), Strasbourg-Kehl (kp 289) and Iffezheim (kp 334). The Kembs-Efringen-Kirchen site is located along the upper stretch of the Upper Rhine study area (Fig 1) and was a braided area before modifications and development that took place in the 19th century. The hydroelectric power station dates from 1932 when the Grand Canal d’Alsace was completed. A large part of the study area is included in the “Petite Camargue Alsacienne” natural reserve. Further downstream, the Strasbourg-Kehl site is located along an historically braided and anastomose sector [12, 13]. The hydroelectric plant here was completed in 1970 and resulted in the creation of an artificial island bounded by a residual part of the Rhine on the east and a channel conducting water to the power plant on the west (known as a ‘festoon layout’). This area is much larger than the others as it fully covers the artificial island (6 km long, including the Rohrschollen nature reserve) and two important bridges in terms of traffic and connection. The Beinheim-Iffezheim site is located in a sector historically comprised of anastomoses and incipient meanders. The Iffezheim hydroelectric plant (completed in 1977) is an online dam (i.e. with no river diversion) and the study area is not subject to national or regional natural reserve regulations.

**Fig 2:**
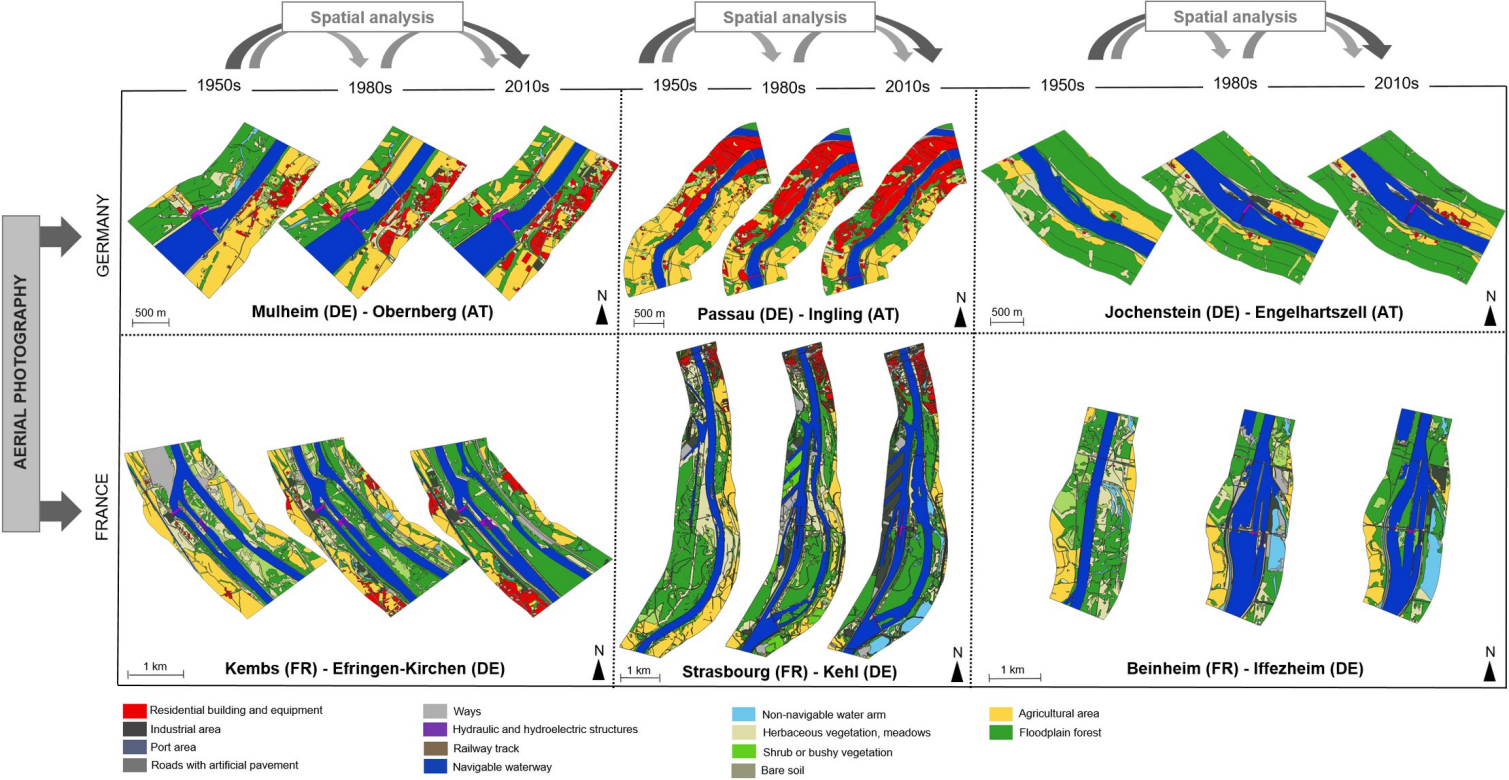
Methodological approach with digitalizations of all six sites based on typology for the three reference dates (1950, 1980 and 2010).

The three sites on the Inn/Danube system are located at Mullheim-Obernberg, Passau-Ingling and Engelhartszell-Jochenstein, all within a radius of 30 km around the 2225 rkm (river kilometric from the mouth of the river) (Fig 1) and are somewhat smaller in area than those on the Upper Rhine (Fig 2). While road and rail bridges are present, the sites are not as economically dynamics as the Rhine sites, in particular compared to Strasbourg-Kehl. The Mullheim-Obernberg site and the Passau-Ingling site are both located along the River Inn, a large tributary of the Danube that feeds a large amount of water from the Alps during the snow-melt period. The Inn is much shallower than the Danube, with an average depth of 1.9 and 6.8 metres, respectively. The Passau-Ingling site is heavily urbanized, owing to the fact that it crosses the city of Passau, and includes several bridges. This section is also navigable and heavily used by cruise ships. The most downstream site is located near the cities of Engelhartszell (Austria) and Jochenstein (Germany). The Jochenstein hydropower plant was built between 1952 and 1956.

All six sites comprised a 500 metre wide buffer strip of land lateral to the main channel of the river, defined from the most recent aerial photographs (Fig 2), our aim being to integrate the diversity of land use and infrastructure networks along the main channel of the hydrosystem. The longitudinal area studied at each site was adjusted to take account of the density of its infrastructure network, with bridges integrated as longitudinal landmarks and at least 500 m of river bank assessed on each river side to ensure the study area included some roads. In this way, the sociological part of the study (impact of transport infrastructure) overlaps with the study area used for the ecological study. When the bridge coincides with a lock or dam along a derivation canal, we used a linear stretch of one kilometre upstream and downstream of the festoon. As regards the Upper Rhine, the Beinheim-Iffezheim site includes a bridge on the hydroelectric dam; the Strasbourg-Kehl site extends 10.4 kilometres from the Pflimlin bridge at its upstream point to the Kehl bridge downstream. The festoon layout at Rohrschollen Island is 800 m wide, making this study area much larger than the others (island width + fairway width + two lateral buffer strips of 500 m). We assessed the development of relict floodplain forest as a cumulative effect of infrastructure networks rather than single structures.

### Spatial analysis and statistics

#### Aerial photography analysis

Evolution of land use was assessed by photo-interpretation using the ArcGIS software at three dates: 1950s, 1980s, 2010s (Fig 2). To achieve this goal, we actively researched appropriate aerial photographs for the defined study area. Historical data were obtained from the Landesamt für Digitalisierung, Breitband und Vermessung. Then, a georeferencing network was performed for each date using homogeneously distributed link points to obtain an average squared error of less than 1. This approach allows for high precision reads with offsets of < 2m. Each sector was then fully digitalized from digitized and geo-referenced raster maps. Any object on the map was vectorised by the creation of polygons in order to perform spatial analyses. We used existing land-use databases, such as BdOCS CIGAL (at a regional level for France) and the Corine Land Cover database (available at a European level) as complementary resources to identify the different types of land use.

The digitization scale was set at 1/800^th^; however, it was sometimes necessary to use a different scale when assessing the oldest photographs in order to distinguish the different types of land use. Fifteen classes were used to define land-use classes (Fig 2): 1) Residential buildings and facilities (e.g. stadiums, sports halls), 2) Industrial areas (e.g. factories, power plants), 3) Port areas, 4) Roads with artificial pavement, 5) Ways (e.g. paths, roads), 6) Hydraulic and hydroelectric structures (e.g. locks, dams), 7) Railway tracks, 8) Navigable waterways, 9) Non-navigable water arms, 10) Herbaceous vegetation and meadows, 11) Shrubs or bushy vegetation, 12) Bare soil, 13) Agricultural areas (e.g. open field or orchard), 14) Tree vegetation, and 15) Mixed vegetation (trees with gaps). The latter two classes were difficult to distinguish in several aerial pictures; hence, they were merged in order to characterize relict floodplain forest surface changes.

#### Spatio-temporal analysis

We analysed land use and the expansion of infrastructure networks for each period and site by aerial photography analysis in land use over time with transition matrices and spatial metrics to describe the importance of the infrastructure networks (Fig 2). To analyse spatial evolution, the land occupation vector layers were rasterized. The rasterization stage makes it possible to use geographical and mathematical tools based on transition matrices that quantify changes in land use between two dates [14]. In our case study, these tools were used to characterize changes in forest area over time, i.e. to clearly quantify the percentage of transition from forest to artificial types of land use such as roads or buildings. Calculations were made to quantify the loss of information between a rasterization at a resolution of 1, 2 and 3 m with the cell size fixed at 3 m a side in relation to the minimum width of the paths and roads (rasterization from the centre). Paths and roads with a smaller width were barely perceptible and classified as pedestrian ways with a negligible effect on the floodplain forest. Three transition matrices were produced, the first calculated for the whole site (global) and then one for each bank separately in order to assess potential differences in the dynamics related to the management practices of each country.

A large number of spatial metrics were used to describe land use changes over time related to, each date; land area, floodplain forest or agricultural area; the linear density of communication channels (km of roads per km of river line); and landscape fragmentation (i.e. the perimeter-area ratio and the number of patches). Calculation of a multiplier coefficient (Cm), a ratio between the most recent date from 2010 and the earliest date considered (1950s), made it possible to quantify the evolution of a metric over the study period. A value of 1 indicated no change, a value <1 indicated a decrease and a value >1 an increase.

## Results

### Changes in land use over time

**Fig 3:**
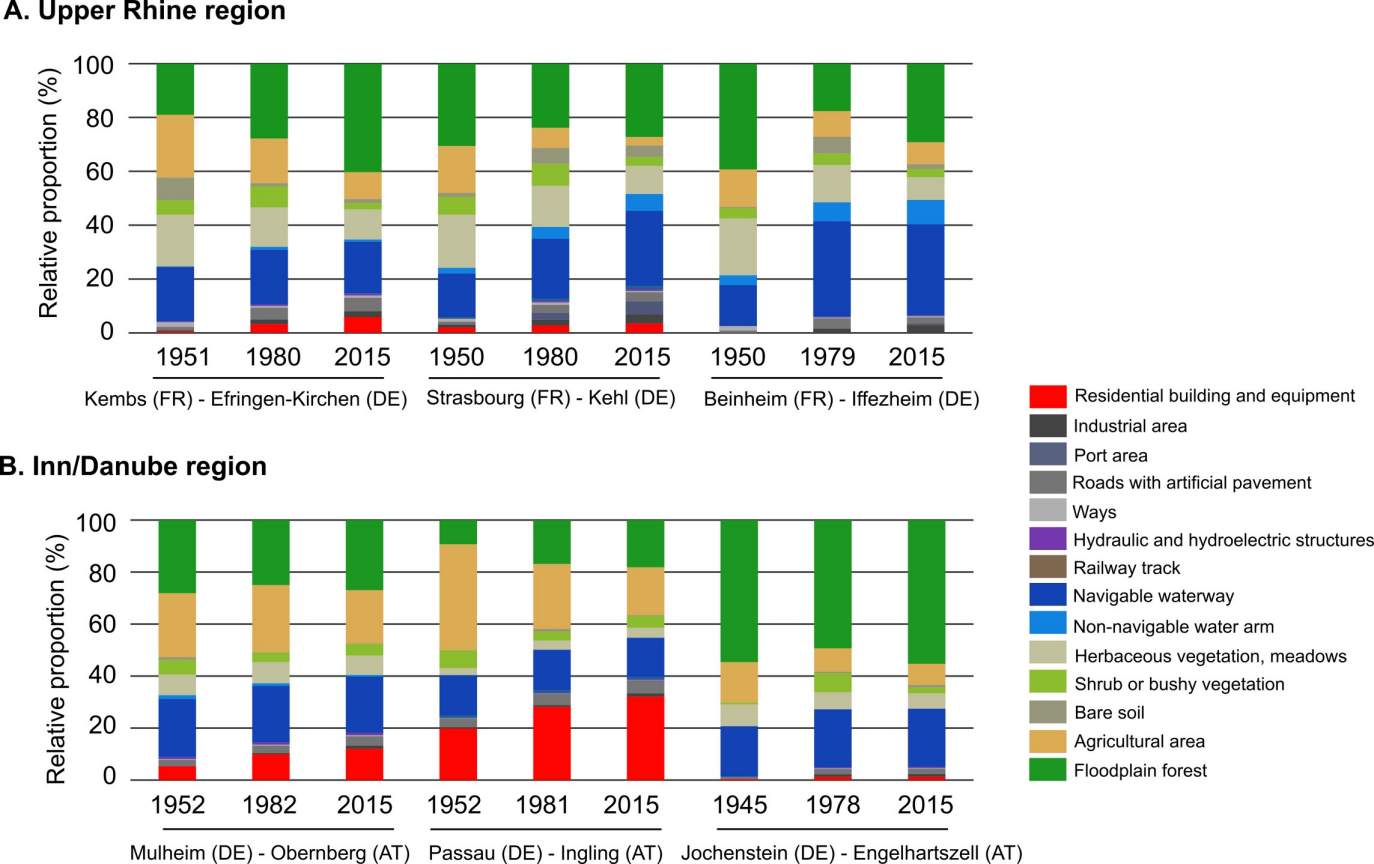
Changes in land cover type on each site for the three periods studied.

Overall, floodplain forest (mixed and tree vegetation) ranged between 9.3 and 55.3%, and agricultural land between 3.1 to 40.7% (Fig 3, S1 Table). The Beinheim-Iffezheim site (Upper Rhine) and the Engelhartszell-Jochenstein site (Inn-Danube) showed a different pattern from the other sites, with most of the area covered by natural habitat types such as herbaceous vegetation, shrub or bushy vegetation, floodplain forest (Fig 3, S1 Table).

**Fig 4:**
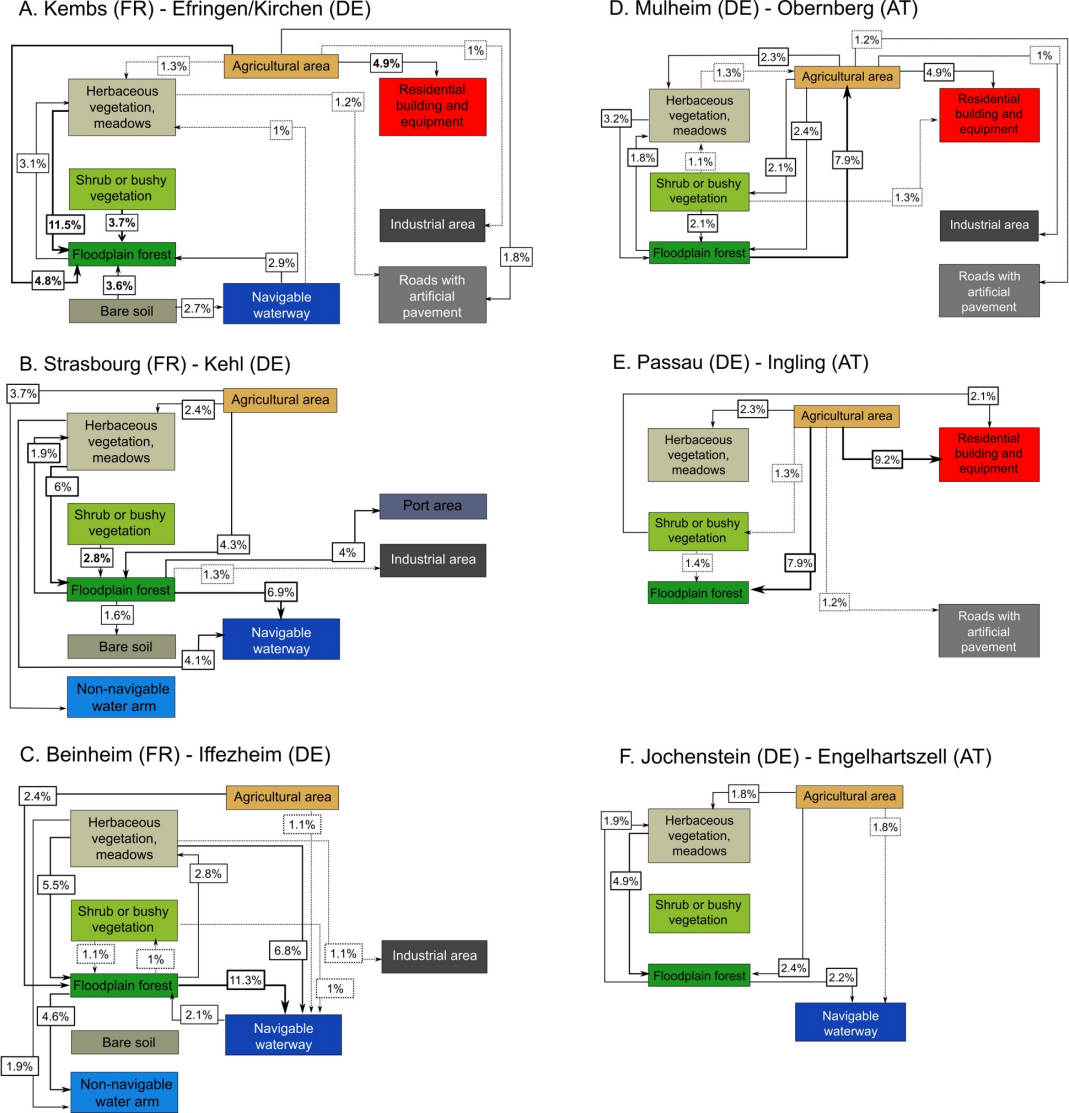
Graphical visualization of global transition matrices covering the 1950s and 2010s for each study site and both regions. A-C = Upper Rhine, D-F = Inn-Danube. The colours of each box are in accordance with the land cover types in Fig 2.

Three sites (Strasbourg-Kehl, Mulheim-Obernberg, Passau-Ingling) showed distinct changes over the study period related to the spread of urban, industrial and harbour zone areas. The two downstream Rhine sites (i.e. Kembs-Efringen-Kirchen, Strasbourg-Kehl) in particular show marked changes due to the installation of hydraulic and hydroelectric installations (Fig 3). On the other hand, forest area increased between the 1950’s and 2010’s at the Kembs-Efringen-Kirchen site (18.9% to 40.2%), the Passau-Ingling site (9.3% to 18.2%), and has declined only slightly at the Beinheim-Iffezheim site (39,2% to 29,2%) (S1 Table). Transition matrices highlighting the main land cover changes between the 1950s and 2010s indicated a decrease in agricultural area over time, and an increase in urban sprawl and forest area (Fig 4). The surface area of shrub or bushy vegetation has also decreased as these usually represent transitional bare surfaces neglected by humans that later develop into forest (Fig 4). While total forest area sometimes appeared to remain constant over time, there were cases where the forest changed location, e.g. where one habitat type has developed into forest while a similar area of forest changed to another habitat type. We calculated that an average of 12.5% of the forest area had changed location, ranging from a minimum of 7.3% at Engelhartszell-Jochenstein to a maximum of 26.5% at Kembs-Efringen-Kirchen (Fig 4).

**Fig 5:**
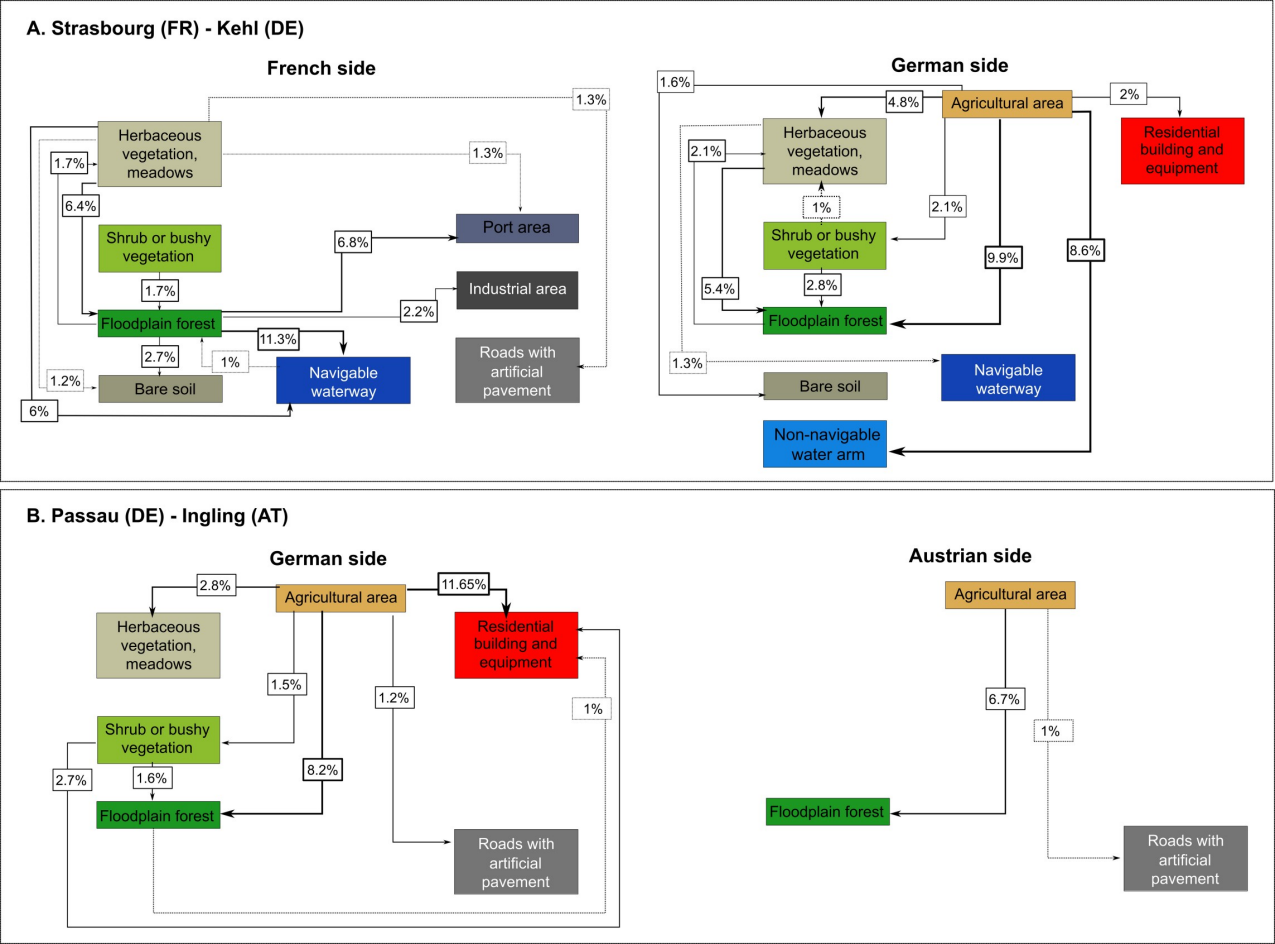
Visualization of transition matrices for the 1950s and 2010s for each country on opposite banks of the river. A. the Strasbourg-Kehl site (Upper Rhine region), and B. the Passau-Ingling site (Inn-Danube region).

Major differences were observed in transition matrices for each bank at each site (Fig 5). As in the other sites, the transitional matrix of the Strasbourg-Kehl site (Upper Rhine) showed a shift away from agricultural use to forest (4.3%) and non-navigable waterways (3.7%). However, matrices for each bank/country showed that the agricultural abandonment occurred on the German side only (9.9% transformed to floodplain forest, 8.6% to non-navigable water arms and 4.8% to herbaceous vegetation). Likewise, there was also a strong transition from herbaceous vegetation to tree vegetation (5.4%). Transition values differed considerably on the French side, where forest area has been reduced through the construction of harbour facilities (6.8%) and navigable waterways (11.3%). In comparison, while country matrices for the Passau-Ingling site also showed a decrease in agricultural area on both sides of the border in favour of floodplain forest (German side = 8.2%, Austrian side = 6.7%), a large part of the agricultural area was transformed into an artificial built area on the German side (11.7%) but not the Austrian side. The transitions observed at the Kembs-Efringen-Kirchen site mainly concerned changes from herbaceous vegetation to trees (French side = 10.2%, German side = 14.4%) and shrubs (3.6% and 4.0%, respectively), with agricultural areas transforming into forest (German side = 9.6%, French side = 2.6%) and built areas (French side = 6.2%, German side = 1.9%). Land use transitions on the German side of the Mulheim-Obernberg site (Inn-Danube), however, showed a reverse dynamic, with 16.6% of forest area transformed into agricultural land, but counterbalanced by a 4.9% transformation of herbaceous vegetation into trees. In comparison, agricultural area on the Austrian side had been transformed into herbaceous vegetation (3.9%), shrub or bushy vegetation (3.7%), forest (4%) or built areas (8.8%). The Beinheim-Iffezheim site was the most “natural” and there was little difference in the levels of transition on either bank. Overall, non-navigable waterways had expanded at the expense of forest (French side = 11.8%, German side = 10.8%), while herbaceous vegetation had gradually transformed into forest (French side = 4.4%, German side = 6.5%). At the Engelhartszell-Jochenstein site, natural land use has persisted over time on the Austrian side, with a slight loss of agricultural area in favour of trees (4.3%). On the German side, agricultural area had also decreased slightly, but in favour of navigable waterways (+2.27%), industrial areas (+1.80%), herbaceous vegetation (+2.02%), built areas (+1.16%) and transport infrastructure (+1.14%). Overall, the infrastructure network had increased over time at all sites except Beinheim-Iffezheim (Table 1; Cm range = 1.33 to 2.62).

**Table 1:** Evolution of five metrics using the multiplying coefficient (Cm) between the most recent (2010s) and oldest date (1950s).

	Forest Area	Agricultural area	Infrastructure network area	Number in forest patch	Number in agricultural patch	Ratio P/A Forest	Ratio P/A Agricultural
**A. Upper Rhine region**							
Kembs-Efringen-Kirchen	2.11 ↗	0.43 ↘	1.90 ↗	1.64 ↗	0.86 =	1.37 ↗	1.45 ↗
Strasbourg-Kehl	0.90 =	0.18 ↘↘	1.80 ↗	1.97 ↗	0.57 ↘	1.32 ↗	0.91 =
Beinheim-Iffezheim	0.74 ↘	0.58 ↘	1.07 =	0.91 =	0.82 ↘	1.05 =	0.46 ↘
**B. Inn-Danube region**							
Mulheim-Obernberg	0.95 =	0.83 =	1.51 ↗	2.18 ↗	0.68 ↘	0.99 =	0.43 ↘
Passau-Ingling	1.95 ↗	0.45 ↘	1.33 ↗	2.33 ↗	0.69 ↘	1.01 =	0.51 ↘
Engelhartszell-Jochenstein	1.01 =	0.52 ↘	2.62 ↗↗	1.06 =	1.55 ↗	1.98 ↗	1.40 ↗

Ratios P/A correspond to the Cm applied to the ratios of perimeter to forest area or agricultural patches.

### Focus on the relict floodplain forest

While forest area has remained relatively stable over time at the Strasbourg-Kehl, Mulheim-Obernberg and Engelhartszell-Jochenstein sites, it has increased at the Kembs-Efringen-Kirchen and Passau-Ingling sites (Table 1). As such, the Beinheim-Iffezheim site is the only one where forest area has actually decreased (Cm = 0.74). On the other hand, all sites were marked by a decrease in agricultural area (Table 1).

**Fig 6:**
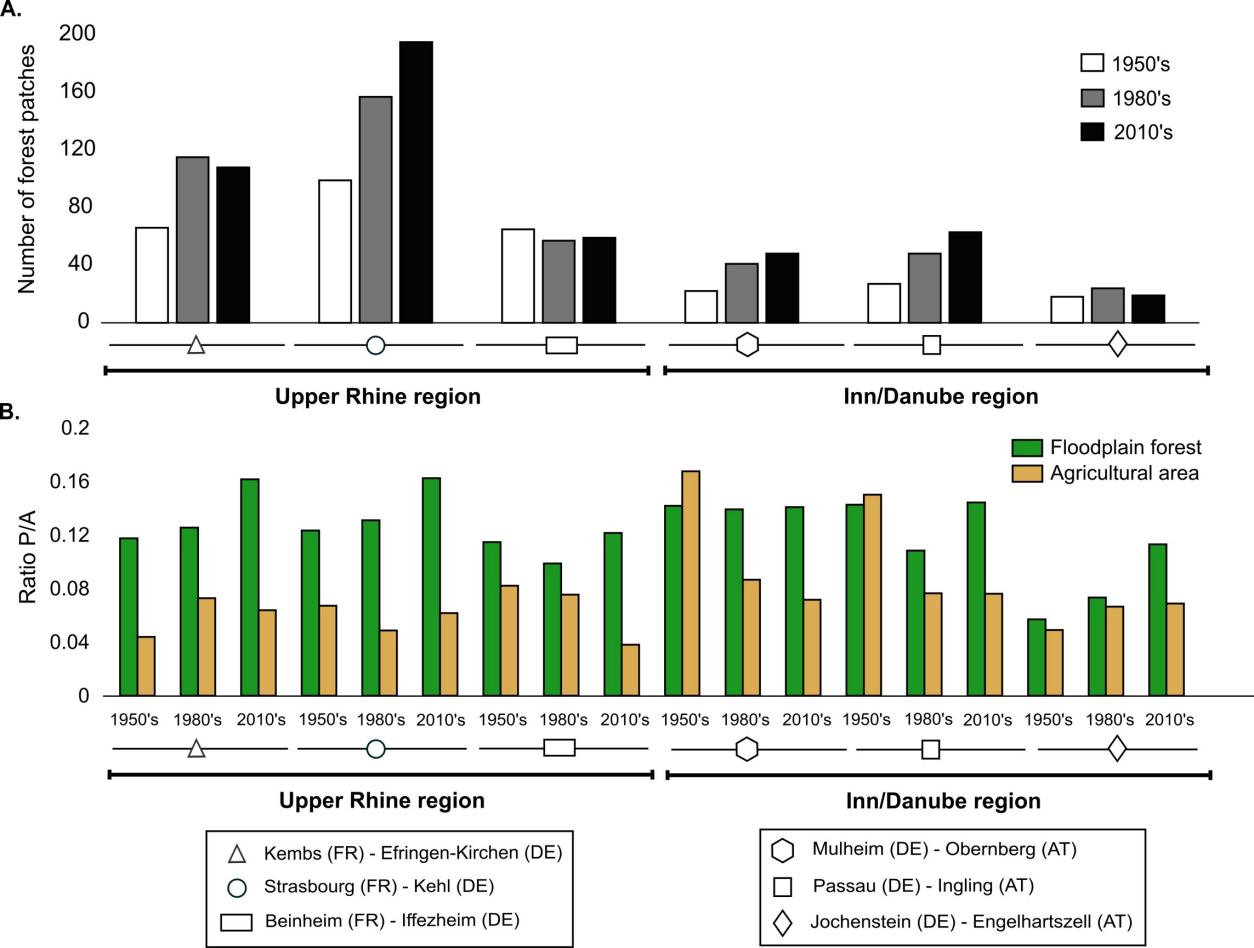
A. Number of relict floodplain forest patches for each study site and each time period; B. Forest and agricultural patch complexity, with the average Perimeter/Area ratio for patches of the same habitat type.

Habitat fragmentation was evaluated using two separate metrics, patch number and the ratio of perimeter to surface area (ratio P/A; Table 1). Patch number indicated that, apart from Beinheim-Iffezheim (no change) and Engelhartszell-Jochenstein, forest area fragmentation has increased overall (Fig 6A and Table 1). While Cm values were high at Mulheim-Obernberg and Passau-Ingling (Cm > 2), Engelhartszell-Jochenstein displayed low fragmentation and a low number of forest patches, with agricultural area increasing over time (Cm = 1.55) but decreasing elsewhere. Overall, there had been an increase in the area covered by linear transport infrastructure (e.g. roads, rights-of-way and railway tracks), especially at the Engelhartszell-Jochenstein site.

Overall, the simplest polygon shapes gave the lowest P/A ratio. Lowest forest area fragmentation was observed at Beinheim-Iffezheim (Rhine) and Engelhartszell-Jochenstein (Inn-Danube), based on both patch number and the P/A ratio for forest patches (Fig 6B and Table 1). At Strasbourg-Kehl and Mulheim-Obernberg, stagnation of forest surface area over time, coupled with an increase in patch number and P/A ratio, revealed an increase in fragmentation over time. At both sites, the patches had become smaller and, at Strasbourg-Kehl, the patch shapes were more complex (Cm = 1.32 vs. Cm = 0.99 at Mulheim-Obernberg). At Kembs-Efringen-Kirchen, forest area has increased (Cm = 2.11), as well as the number of patches (Cm = 1.64) and the P/A ratio (Cm = 1.37). The Passau-Ingling site showed a gain in forest area similar to that at Kembs-Efringen-Kirchen (Cm = 1.95) and, while patch shape has remained substantially unchanged (Cm = 1.01), the number of patches has increased sharply (Table 1).

## Discussion

### Land use changes: Statement of the area occupied by the floodplain forest

Changes in land use and the expansion of infrastructure networks have had a clear influence on the relict floodplain forest along the six river stretches studied on the Rhine and Danube/Inn rivers. Through the use of transition matrices, it was possible to highlight transitions between land use types between the 1950s and 2010s. One striking result of this study was that the overall (global) surface area of relict floodplain forest at these sites has not decreased since the 1950s (except for a small area at the Beinheim-Iffezheim site); indeed, at two of the study sites, the forest surface area has actually increased. All of the study sites, however, represented border zones between two countries and there was potential for differences based on national management regimes. Along the Upper Rhine, for example, many of the habitat changes on the French side were the result of natural evolution of unmanaged areas, while agricultural abandonment was the main factor on the German side. Field inventories should make it possible to know how the plant communities of these environments with different heritages have evolved. Along the Inn/Danube region, on the other hand, the increase or stagnation of floodplain forest areas was clearly attributable to agricultural abandonment on both sides of the river.

While the global forest area had not decreased, or had remained roughly of the same order of magnitude (Table 1), we observed major land use changes locally. Parts of the 1950s forest had been transformed into other habitat types, but this was counterbalanced by the conversion of other habitats into forest, either by natural development of pioneer habitats or through the effects of agricultural abandonment. Hence, parts of the area qualified as forest actually correspond to patches of a young, recent established ecosystem. The changes that have occurred between the 1950s and 2010s (average 12.5%, range 7.3%– 26.5%) have led to an increased potential for forest area fragmentation alongside fragmentation caused by the linear infrastructure network (road, rail, etc.).

### A patchy green corridor

In most cases, the fragmentation caused by development of transport infrastructure has produced a patchy green corridor whose functionality and interfaces deserve further study if there are to be improvements. Functions of the floodplain forest include support services such as breeding areas, migration corridors, but also ecological processes such as a reduced soil erosion or the improvement of water quality [15]. While the forest area has increased at most sites, or at least remained stable, patch fragmentation and the forest edges remain a crucial issue in terms of quantity and quality [16]. We clearly observed fragmentation of the relict floodplain forest in four of the six sites studied, and a decrease in forest patch interfaces favourable for biodiversity in five of the six sites. A patchy environment implies small sub-populations more prone to local extinction, making the forest buffer zone and other habitats more important over time. A higher number of patches also implies that the contacts between natural and artificial habitats or linear infrastructure become more important than those between forest patches and other natural habitats [17].

One way to compensate against the need for large roads related to economic development is to build them at locations where small roads already exist [16]. Though this strategy has been partly applied in our study areas, our results showed that the linear infrastructure network has tended to develop at the expense of natural habitats. These artificial networks encourage social and economic relationships between anthropic habitats (agricultural fields, urban areas, etc.) that have grown up in the vicinity of relict floodplain forest patches. As a consequence, the landscape has become patchier (rougher) and no longer provides free and safe movement of species [17]. As such, forest patch edge effects have become more and more important over the period studied.

### The weight of the legacy of sites on the conservation of the floodplain forest

While our results were congruent for most of the sites investigated, two things must be kept in mind: 1) while our study period was relatively recent, profound changes also took place at the sites prior to the 1940s, and 2) the spatial buffer implemented along our river sites was consistent with land use today; however, the natural floodplain was very much larger in the past [12]. The spatial areas considered in this study comprised a 500 metre wide strip of land running up- and downstream at each site. Even two centuries ago, this width was mainly covered with floodplain forest and secondary arms both in the Upper Rhine and the Inn/Danube rivers. Territorial development over the last two centuries had modified the landscape along the river profoundly, well before the earliest period considered in this study (the 1940s). On the Upper Rhine, for example, three successive phases of major engineering works (channelization, groyne construction and damming) had affected the river’s structure [9]. Channelization was performed between 1842 and 1876 and this major development fixed the main channel of the river; indeed, the current topographic gradient has been inherited from these early engineering works. The loss of active floodplain and the transformation of gravel bars and islands into vegetation or agricultural land following channelization can be observed all along the Upper Rhine continuum, from Basel to Lauterburg [18]. Here, authors recorded a 93% reduction in fully connected channel area and a 513% augmentation in the area of disconnected water bodies over a 97-year period [9]. Indeed, the Upper Rhine has probably changed much more over a century than the Austrian Danube in the Austrian Machland region has over a 179-year period, with a 65% reduction in fully connected channels whereas disconnected water bodies (backwaters) doubled along a 10.25-km-long reach [19]. During the last two centuries, 80% of the French side of the Upper Rhine have been modified through loss of riparian areas and wetlands in favour of urban, agricultural and harbour areas [12, 20]. Like the Upper Rhine, the upper part of the Danube has also undergone major developments that have caused a significant loss of alluvial forest [21], with only 5%, 25%, 28% and 70% of the original alluvial forest remaining in the upper, middle, lower and delta stretches, respectively.

Without river flooding, the only option for maintaining relict floodplain forest in these areas is to ensure spatial planning that fulfils multiple ecosystem services [3, 22]. Riparian forests of fluvial systems are hybrid ecotones in that they result from co-construction by human and natural processes [4, 23]. The ecological, economic and social values of the forestry bordering rivers is today widely recognized [15]. However, in practice, the implementation of sympathetic management in these forest environments remains difficult without indicators reflecting changes in land use. To go further, we have to consider these systems as socio-ecosystems, and we must also consider the viewpoints of the different stakeholders involved in spatial planning of environments bordering large rivers, including infrastructure operators, municipalities and other user groups. Thus, future management of these relict forests should take full account of both current and historical practices related to infrastructure operation and usage, the role that biodiversity plays for stakeholders, and the interdependency between these factors.

## Conclusions

This work is an international and multi-disciplinary study exploring the interactions between the relict floodplain forest and land use. It assesses the influence of land use changes and expansion of infrastructure networks on the relict floodplain forest along the Upper Rhine and the Danube, the oldest and most important trade routes in Europe. Over the past 200 yrs., these fluvial stretches have been strongly shaped by structural modifications such as channelization, hydropower plants, locks, railway crossings and roads. One striking result of the study was that, though we observed major land use changes on a local scale, global forest area had not decreased but remained roughly of the same order of magnitude over the years. Parts of the 1950s forest had been transformed into other habitat types, but this was counterbalanced by the conversion of other habitats into forest, either by natural development of pioneer habitats or through the effects of agricultural abandonment. However, both ecological changes and changes in the linear infrastructure network (road, rail, etc.) between the 1950s and 2010s have led to an increased forest area fragmentation. Without river flooding, the only option for maintaining relict floodplain forest in these areas is to ensure spatial planning that fulfils multiple ecosystem services. Our study provides a method for obtaining indicators reflecting changes in land use over time that allow the implementation of rational management in these relict floodplain forests at an international level. Now, we would explore more accurately the fragmentation and new borders caused by development of transport infrastructure. It has produced a patchy green corridor whose state of conservation should be verified in the field. The future of this relict floodplain forest will be also characterized depending on the evolution of uses in accordance with a regulation of habitat conservation.

## Supporting information

S1 Table Raw data as basis for spatial analyzes. The percentages of land use (%) are available for all six sites over time.
(XLSX)
